# Preparation and Properties of Nanostructured GaN-Reinforced Cu Matrix Composites

**DOI:** 10.3390/ma18112489

**Published:** 2025-05-26

**Authors:** Yunlong Bai, Hui Ge, Yaoyang Peng

**Affiliations:** 1 Key Laboratory for Ecological Metallurgy of Multimetallic Ores (Ministry of Education), Northeastern University, Shenyang 110819, China; baiyl@smm.neu.edu.cn (Y.B.); 1810547@stu.neu.edu.cn (Y.P.); 2 School of Metallurgy, Northeastern University, Shenyang 110819, China; 3 Key Laboratory for Recycling of Nonferrous Metal Resources, Northeastern University, Shenyang 110819, China

**Keywords:** GaN-Cu, copper matrix composites, mechanical alloying, powder metallurgy

## Abstract

As a pioneering exploration of gallium nitride (GaN) as reinforcement in metal matrix composites, this study systematically investigated the mechanical–electrical property evolution in copper matrix composites through controlled GaN incorporation—a research gap scarcely addressed previously. GaN-Cu composites with tailored GaN contents were successfully synthesized by precisely controlled mechanical alloying and powder metallurgical processing and exhibited exceptional mechanical–electrical synergies. Advanced microstructural characterization via X-ray diffraction and electron microscopy revealed the homogeneous dispersion of GaN nanoparticles within the Cu matrix, forming coherent interfacial structures. The characterization results show that GaN-Cu composites could be successfully prepared by mechanical alloying and powder metallurgy methods, and it was confirmed that GaN nanoparticles could improve the mechanical properties of metal matrix composites as reinforcement; with an exponential increase in GaN content, the decrease in conductivity became very slow. With an increase in GaN content, the electrical conductivity decreased in an “L” shape, while the hardness first increased and then decreased, but the hardness could reach up to 128.66 HV, which is about 130% higher than that of the substrate.

## 1. Introduction

Copper matrix composites have a higher specific modulus, higher specific strength, higher hardness, better wear resistance, and excellent electrical conductivity compared to unreinforced copper metal [1,2,3,4,5]. Copper, copper alloys, and Cu-based composites are widely utilized in advanced industries due to their exceptional electrical conductivity, thermal management capabilities, and mechanical strength. In aerospace, they enable lightweight electrical systems and thermal control for spacecraft components. The military sector employs ceramic-reinforced Cu composites in armor and weapons for enhanced durability, while automotive applications focus on high-efficiency wiring and heat exchangers. Electronics leverage copper’s conductivity for miniaturized circuits and PCB interconnects, and nuclear/energy industries rely on their radiation resistance and corrosion stability. These multifunctional properties make them indispensable across cutting-edge technological domains demanding material versatility [6,7,8]. To further enhance the comprehensive properties of CuMCs, various reinforcement materials such as Al_2_O_3_, SiC, ZrO_2_, and graphene have been extensively studied and used [9,10,11,12,13,14,15,16].

Gallium nitride (GaN) is a wide-bandgap semiconductor with excellent electrical properties and high hardness, which plays an important role in modern blue light technology and energy-saving light-emitting diodes with a stable hexagonal lattice crystal structure [17,18,19]. GaN and silicon carbide (SiC) are both considered third-generation semiconductors [20,21,22]. Based on the reported data, SiC has been used to enhance the strength of the copper matrix; to our knowledge, few studies are available on GaN-Cu metal composites developed by the powder metallurgy method. Drory et al. determined the mechanical data of GaN, and they pointed out that the Young’s Modulus and Vickers Hardness of GaN reached 287 GPa and 12 GPa, respectively [20]. Mariusz et al. reported that they had successfully made gallium nitride bulk by sintering pure gallium nitride nanopowders at 900 °C, 6 GPa, and 310 min, with an excellent hardness of 13 GPa [21]. Some of the unique properties of GaN such as high hardness, a low coefficient of friction, chemical inertness, and a high melting point seem to herald the possibility of GaN-Cu composites.

Various techniques are employed to produce reinforced copper matrix nanocomposites, which can be broadly classified into two major groups, namely, liquid metallurgy (LM) and powder metallurgy (PM). Liquid metallurgy is based on the formation of reinforcing particles in situ in the matrix through chemical reactions or phase changes [23,24]. Meanwhile, the powder metallurgy method usually uses the mechanical alloying or selective crushing of secondary particles to mechanically disperse the reinforcement into the matrix [25,26]. Considering the chemical stability of GaN, it is more difficult to prepare GaN-Cu composites by the liquid metallurgy method. Hence, the powder metallurgy method may be a more feasible method for preparing GaN-Cu composites. This can not only uniformly mix Cu and GaN powders and refine Cu and GaN particles but also store a huge amount of energy on the surface of the particles to promote the combination of the two [27,28].

Despite these advantages, challenges remain in optimizing the GaN-Cu interface and balancing mechanical–electrical performance. For instance, excessive GaN content may lead to agglomeration, reducing both mechanical strength and conductivity. Additionally, the mismatch in thermal expansion coefficients between Cu (17 × 10^−6^/K) and GaN (5.6 × 10^−6^/K) could induce residual stresses during thermal cycling, potentially affecting long-term reliability [29]. Addressing these challenges requires a systematic investigation of processing parameters (e.g., milling time, sintering temperature) and their effects on microstructure–property relationships. Previous studies on SiC-Cu composites have demonstrated that reinforcement morphology and interfacial bonding play critical roles in determining composite performance [30,31,32]; however, analogous insights for GaN-Cu systems remain scarce. This knowledge gap highlights the need for focused research to unlock the full potential of GaN as a multifunctional reinforcement in copper matrices.

In this study, the mechanical and electrical properties of GaN-reinforced copper-based composites prepared by powder metallurgy were systematically investigated, and the purpose of this study was to explore the effect of controlled GaN doping on the microstructure and properties of composite materials. By employing advanced microstructural characterization techniques such as X-ray diffraction and electron microscopy, the uniform dispersion and coherent interface structure of GaN nanoparticles in a Cu matrix were revealed. The research results indicate that GaN nanoparticles can significantly improve the mechanical properties of CuMCs while maintaining reasonable electrical conductivity. This work not only contributes to the fundamental understanding of GaN-Cu composites but also provides insights into their potential applications.

## 2. Materials and Methods

### 2.1. Materials

The raw materials used for mechanical alloying were powders of copper (Cu; 99.99 wt% purity; Yingtai Metal Materials Co., Ltd., Ningbo, China) and gallium nitride (GaN 99.9 wt% purity; Shanghai Macklin Biochemical Co., Ltd., Shanghai, China).

### 2.2. Experiments

GaN-Cu composites with GaN mass contents of 2.5 wt% (2.5 GaN-Cu), 5 wt% (5 GaN-Cu), 7.5 wt% (7.5 GaN-Cu), and 10 wt% (10 GaN-Cu) were designed in this work, and the mixed powder of Cu and GaN was configured according to the corresponding design ratio. Agate balls had diameters of 10 mm and 6 mm and a ball-to-powder weight ratio of 20:1. Ethanol was used as the process control agent (PCA) to reduce the cold soldering of the powder during milling and to prevent the copper powder from adhering to the agate balls and vessel walls. Nitrogen was charged to prevent the oxidation of the Cu powder during the mechanical alloying process. The total powder charge was 8 g, and the speed of the ball milling was 300 rpm. To avoid overheating the powder, the ball milling process was carried out in a non-cyclic mode in planetary ball milling with 15 min stopping intervals every 5 h. The process was repeated three times to ensure a total work time of 15 h.

In order to obtain a large sample, the milled powder was pressed in a steel mold of Φ18 mm at a pressure of 200 MPa and then sintered under a nitrogen atmosphere at 900 °C for 2 h to the preparation of the GaN-Cu block. After the GaN-Cu block was cooled, it needed to be further pressed and sintered to obtain GaN-Cu composites. The GaN-Cu block was cold-pressed with a pressure of 900 MPa and sintered under a nitrogen atmosphere at 900 °C for 2 h. The heating rate and cooling rate for sintering were both 5 °C/min.

### 2.3. Characterization

The crystalline structures and composites of the GaN-Cu samples were determined by X-ray diffraction (XRD) (Cu Kα1, D8 Advance, Bruker, Bremen, Germany). Field emission scanning electron microscopy (SEM) (Zeiss UltraPlus, Duesseldorf, Germany) was used to determine the surface morphology of the milled powders and sintered GaN-Cu samples. Mechanical properties were assessed using a Vickers hardness tester (Wilson Wolpert 402 MVD, Wolpert, Dayton, OH, USA) under a 500 gf load with a 15 s dwell time, averaged over five indentations per sample. Electrical conductivity measurements were performed via a four-point probe system (Pro4-4400, Lucas Labs, Gilroy, CA, USA) in compliance with ASTM F1529 [33], with currents ranging from 1 to 100 mA at ambient temperature (25 °C). Density was quantified using Archimedes’ principle (ASTM B962-15 [34]) via ethanol immersion, while thermal stability was evaluated through thermogravimetric analysis (TGA, STA 449 F5, Netzsch, Selb, Germany) under nitrogen flow (50 mL/min) with a heating rate of 10 °C/min up to 800 °C. These standardized protocols ensured reproducibility and alignment with established material characterization methodologies.

## 3. Results and Discussion

### 3.1. Microstructure and Phase Analysis of GaN-Cu Composite Powder

**Figure 1:**
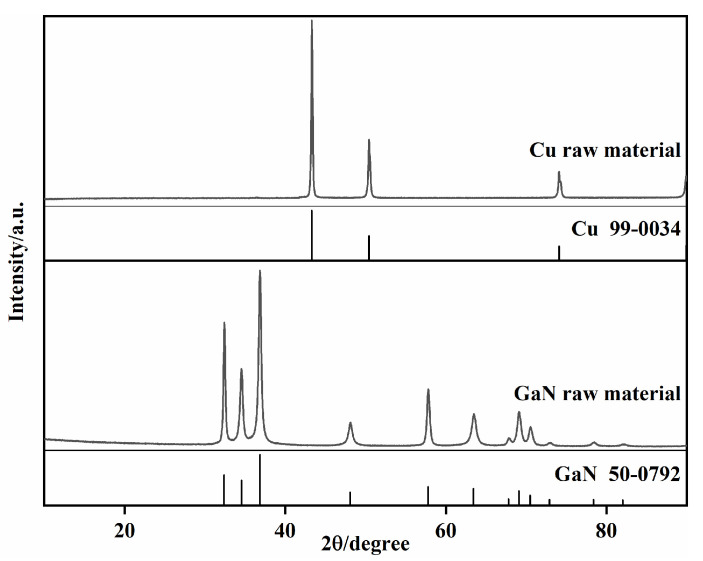
X-ray diffraction patterns of raw material powder of Cu and GaN.

**Figure 2:**
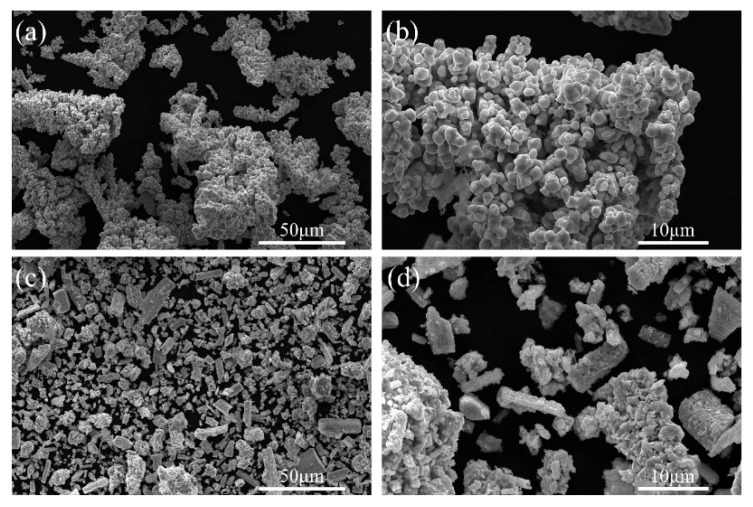
SEM images of raw material (Cu powder (**a**,**b**) and raw GaN powder (**c**,**d**)).

The X-ray diffraction (XRD) and the scanning electron microscope (SEM) characterization of raw materials are shown in Figure 1 and Figure 2, respectively. The XRD results reveal that Cu powder and GaN powder are both standard materials. Figure 2a presents the micromorphology of raw Cu powder and raw GaN powder. Most of the Cu powder exists in the form of irregularly shaped clusters with a diameter of 30–50 μm, as shown in Figure 2a, and the clusters are composed of multiple small particles with a diameter of 0.5 to 1.5 μm, as shown in Figure 2b. GaN powder is typically found in the form of rods, as shown in Figure 2c, with a width of around 10 μm and lengths that vary. These rods consist of many spherical nanoparticles smaller than 1 μm, as shown in the magnification of Figure 2d.

**Figure 3:**
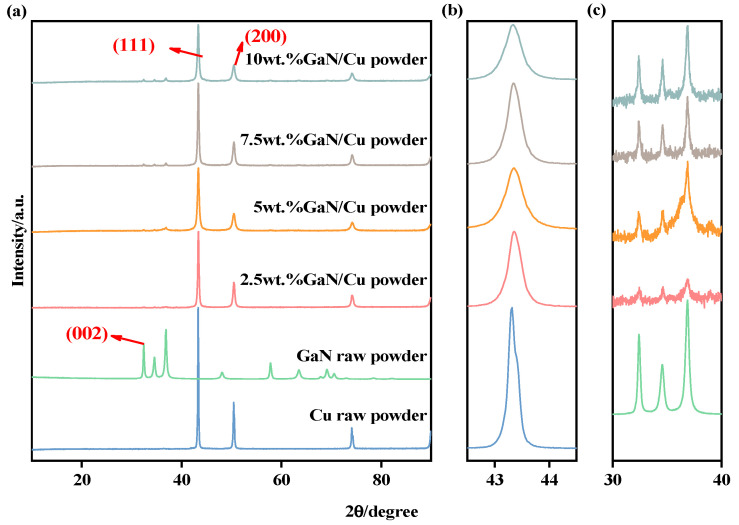
X-ray diffraction patterns of milled GaN-Cu composites powder with different GaN content (**a**) and (**b**,**c**) partial enlarged views.

The phase compositions of GaN-Cu composites powder were studied by XRD analysis, and the XRD patterns are shown in Figure 3. As shown in Figure 3, three obvious peaks located at 43.32°, 50.45° and 74.12° can be observed, which indicate that the main phase composition of the samples is Cu. The three faint peaks located at 32.38°, 34.56°, and 36.85° suggest the presence of GaN. The powder samples formed by mechanical alloying only comprised a combination of GaN and Cu, and neither produced any new material, indicating that mechanical alloying is a viable method for producing GaN-Cu composites. After ball milling, the intensity of the diffraction peaks decreased, such as the diffraction peaks at 43.32°, 50.45° and 74.12°, and the broadening of diffraction peaks was observed, as shown in Figure 3b. The grain refinement in GaN-Cu composites stems from severe plastic deformation during high-energy ball milling, where impact and shear forces generate dislocations and dynamic recrystallization, causing grain subdivision. This structural evolution induces XRD peak broadening through crystallite size reduction (Scherrer equation) and lattice strain accumulation (Williamson–Hall analysis). Concurrently, increased high-angle grain boundary density (∝d^−2^) synergizes with lattice distortions (>0.15% microstrain) to enhance strength via Hall–Petch strengthening (σ_y∝d^−1/2^) and Orowan mechanisms from GaN-induced dislocation pinning [30,31].

**Figure 4:**
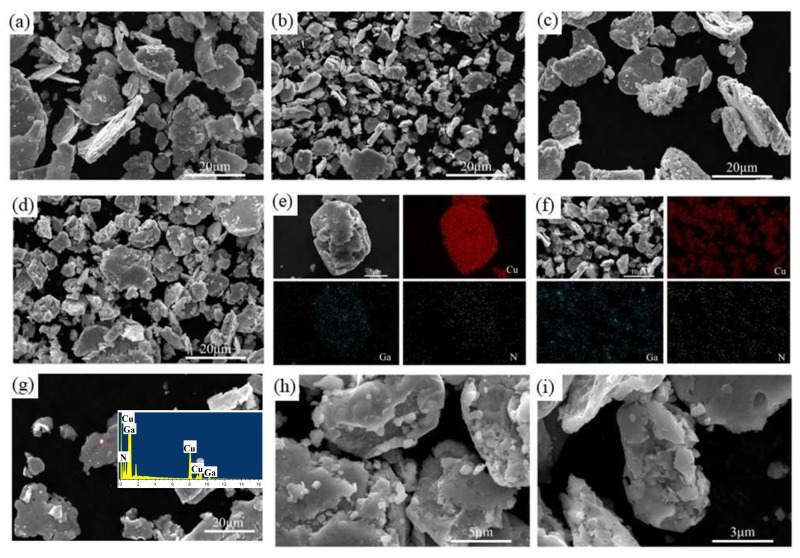
SEM images of powder after ball milling: (**a**) 2.5 wt% GaN-Cu, (**b**) 5 wt% GaN-Cu, (**c**) 7.5 wt% GaN-Cu, (**d**) 10 wt% GaN-Cu. Mapping of 5% GaN-Cu composite powder, (**e**) single particle of 10 wt% GaN-Cu, (**f**) EDS spectra analysis of particles, (**g**) SEM images of 10 wt% GaN-Cu, (**h**,**i**) particles in powder with higher magnification.

The surface morphologies of the mixed powder after ball milling were investigated by SEM analysis, and SEM images of samples are shown in Figure 4. During the mechanical alloying process, the morphology and microstructure of the powder particles change due to repeated deformation, fracture, and welding [28]. Figure 4 shows that after 15 h of ball milling, the particles have various degrees of cracking. The particle diameters of the composite powders with different GaN content were roughly in the range of 0.5–20 m, the particles were independent of one another, and there was no situation of multiple particle bonding as there was in the case of the raw copper powder, which achieved the mechanical alloying goal. It is worth noting that the particles of the composite powder with 5% GaN content appear finer compared to the other components, and this result corresponds to the XRD results, which could show that these experimental ball milling conditions are more conducive to achieving a GaN content of 5%. In addition, it can be seen that the number of irregularly shaped tiny particles with distinct and sharp boundaries increases significantly with the increase in GaN content. The mapping of the mixed powder and the individual particles in the powder after ball milling is shown in Figure 4e,f. The results of the mapping analysis show that ball milling with 15 h not only allows for GaN and Cu to be uniformly mixed in the composite powder but also for each particle in the powder to be dispersed with GaN, and there is no phenomenon in which the same phase material is agglomerated with itself but separated from another material. The high-magnification SEM images of such particles and their EDS analysis are shown in Figure 4g. The results of EDS analysis show that the small particles marked with sharp edges and corners are mainly GaN and Cu. After analysis and assessment, the small particles with sharp edges and corners were determined to be almost certainly made of GaN; this is because GaN grains are harder and more easily to produce small grains with sharp edges and corners than Cu grains. However, the surface is invariably stained with fine nano Cu particles during the ball milling process. Figure 4h shows that in 10 GaN-Cu powder, multiple small GaN particles with sharp edges are embedded on the surface of the composite particles. It can be concluded that the diameter of the GaN particles is about 300–1000 nm, which indicates that the reinforcement has reached the nanometer level. And in the same ball milling time, a lower content of GaN is more favorable to the crushing of GaN particles. Therefore, it can be inferred that the GaN particles in all composite powders reached the nanometer level, which allowed the preparation of GaN-Cu composites at the nanometer level. Figure 4i shows that GaN nanoparticles are embedded not only on the surface but also the interior of the particles.

### 3.2. Microstructure and Phase Analysis of Sintered GaN-Cu Composites

**Figure 5:**
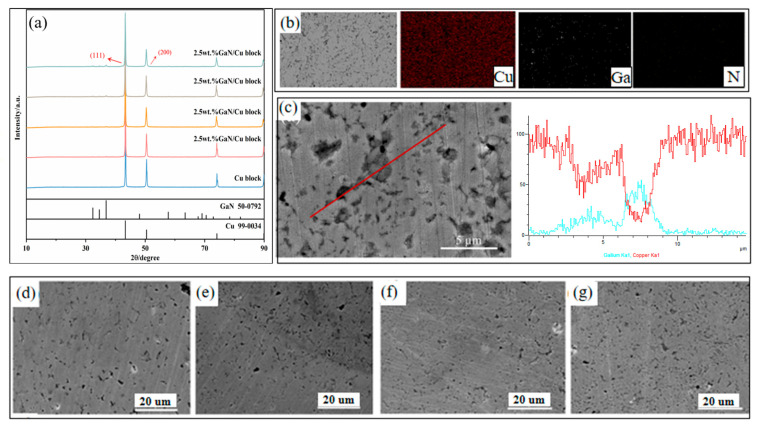
(**a**) X-ray diffraction patterns of sintered GaN-Cu composites with different GaN content, (**b**) mapping of 10 wt% GaN-Cu, (**c**) EDS spectra analysis of 10 wt% GaN-Cu, SEM images of sintered GaN-Cu composites ((**d**) 2.5 wt% GaN-Cu, (**e**) 5 wt% GaN-Cu, (**f**) 7.5 wt% GaN-Cu, (**g**) 10 wt% GaN-Cu).

The XRD patterns of the sintered GaN-Cu composites with different GaN contents are shown in Figure 5a. After repeated sintering, the phase compositions of GaN-Cu composites are still of GaN and Cu, and high-temperature sintering does not generate any additional substances, which is very important when investigating the effect of GaN on the properties of GaN-Cu composites. The peak width of GaN did not change much when compared to the powder XRD before sintering, but the peak intensity of GaN was lowered dramatically, and the Cu peak became sharper, suggesting that the Cu grains increased, while GaN did not change throughout the sintering process. It is noteworthy that the peak width of the sintered copper remained almost unchanged but the peak intensity decreased with the increase in GaN content. The decrease in peak intensity can be attributed to two problems. The first is the different thermal expansion coefficient between the GaN particles in the second phase and the Cu matrix, which leads to the formation of lattice microstrain in the Cu matrix [35]. The second is the reduction in, or refinement of, the grain size. In the case of hybrid nanocomposites, the uniform distribution of nanoparticles in the Cu matrix significantly reduces grain growth and the GaN nanoparticles prevent grain growth by penetrating the Cu grain boundaries and immobilizing them, which leads to fine-grained composites through the Zener mechanism [36,37].

Figure 5b shows the SEM micrograph and corresponding mapping analysis of 10 GaN-Cu, which were performed to determine the distribution of the elements in the structure. The results show that even after high-temperature sintering, GaN was still uniformly distributed in the copper matrix, but the GaN particles adjacent to each other did not aggregate together due to sintering. The homogeneous dispersion of GaN as reinforcement is a prerequisite in improving the mechanical properties of GaN-Cu composites.

Figure 5c shows that the bright gray material is the copper matrix, while the dark gray is the GaN particles, and the black shows the pores of the composite material. It can be seen that the pores rarely appear alone but appear around the GaN, which is caused by the large difference between the expansion coefficient of Cu and GaN during the sintering and heating and cooling processes. The SEM image of the GaN-Cu composites in Figure 5d–g shows that as the GaN content increases, it is accompanied by an increase in the dark gray material around the black, while the black part representing the voids becomes larger but the diameter of individual voids becomes smaller, which is due to the obstruction of copper grains by GaN particles in the sintering growth process with the increase in GaN content. While the diameter of a single GaN particle is about 700 nm to 1.5 μm only, the GaN particles hardly grow. 

### 3.3. Density and Relative Density Analysis of GaN-Cu Composites

**Figure 6:**
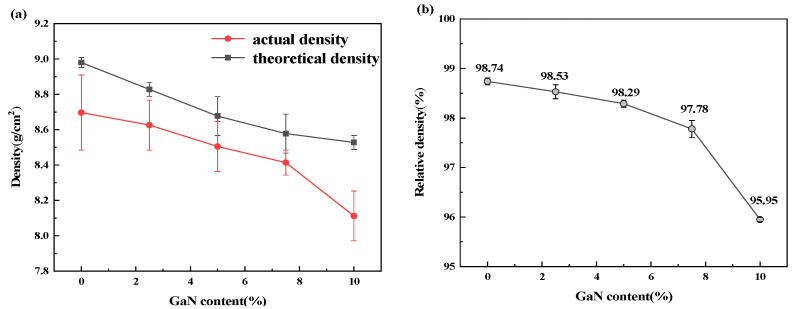
Effect of GaN content on the density (**a**) and relative density (**b**) of the sintered GaN-Cu composites.

The density of the GaN-Cu composites decreases as the GaN content increases because GaN has a lower density than Cu. Overall, the actual density of the GaN-Cu composites is lower than the theoretical density, which is inevitable because there will be voids that inevitably cannot be discharged during the pressing and sintering stages of the powder metallurgical preparation of the material. Figure 6a shows that the decrease rate of density is almost the same when the GaN content is less than 5%, but with the increase in GaN content, the decrease rate of the density is higher and higher. This situation is caused by the fact that the hardness of GaN particles is greater than that of Cu particles and the GaN particles hinder the mutual wedging and hooking between the powders, meaning that the pressed block is looser with the increase in GaN content when the pressing pressure is limited to 900 MPa. For the above situation, boosting the pressure is the right choice. However, for this work, 900 MPa is the maximum pressure that can be provided. On the other hand, the change in relative density will cause a great change to many properties of the composite material prepared by powder metallurgy, and it is no exaggeration to say that density and relative density are among the most important indicators in judging the quality of powder metallurgy products. With the same metal composites, relative density changes have a very strong effect on mechanical properties and electrical conductivity, so, when exploring the effect of the content of a certain reinforcement on the composites, the extreme difference in relative density should be as low as possible. Figure 6b shows that the relative density decreased by 2% when the GaN content increased from 7.5% to 10%, which inevitably may have caused the mechanical and electrical properties to decrease due to the increase in voids, but the relative density of the GaN-Cu composites was still controlled within 3%, and the relative densities of all GaN-Cu composites were higher than 95%, which had reached a relatively high level.

### 3.4. Microhardness Analysis of GaN-Cu Composites

**Figure 7:**
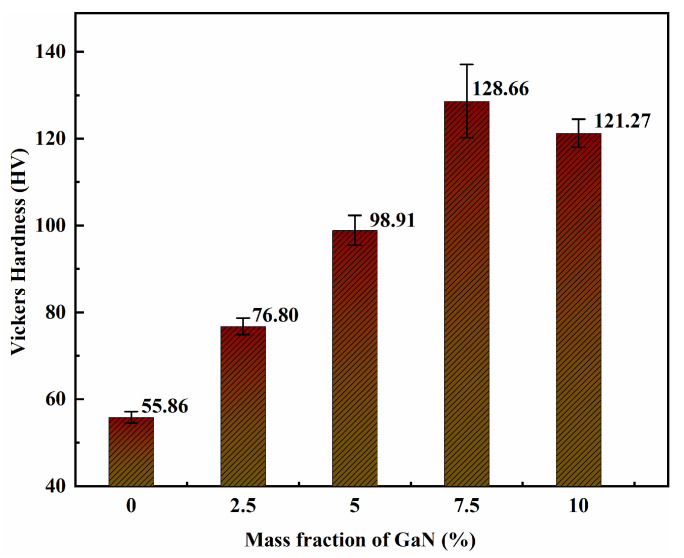
Relation between microhardness and GaN mass fraction of sintered GaN-Cu composites.

The relationship between the hardness values of the sintered GaN-Cu composites and the GaN mass content is shown in Figure 7. The microhardness of the sintered GaN-Cu composites shows a trend of first increasing and then weakly decreasing with the increase in GaN content. The average hardness of pure Cu without the addition of GaN was 55.86 HV prepared under this condition, which is consistent with the hardness range of pure Cu prepared by powder metallurgy. With the addition of GaN, the hardness value increased significantly. When the content of GaN was 2.5% and 5%, the hardness of the composites reached 76.80 HV and 98.91 HV, respectively. At 7.5%, the maximum hardness value reached 128.66 HV, which was 130% higher than that of pure Cu. However, when the content of GaN reached 10%, the hardness of the GaN-Cu composites decreased slightly but still reached 121.27 HV.

The increase in hardness is due to the enhanced diffusion strengthening effect caused by the increase in GaN content. The Orowan mechanism plays a significant role in this strengthening, where the presence of GaN nanoparticles in the matrix causes dislocation rings to remain after the dislocation lines pass through the particles, which also hinders or slows down the dislocation motion in the copper matrix; these processes lead to an increase in hardness. The reduction in microcrystal size by ball milling has a strong influence on the hardening of the composites, especially when the reinforcement size is less than 100 nm [29,30]. In general, with other conditions kept constant, the hardness of the composites increased to varying degrees with the increase in the reinforcement content when the content of the reinforcement did not exceed 20%. A decrease in hardness may only occur with the continued increase in the content when substances with weak interlayer bonding like graphite or graphene nanosheets are present as reinforcements [31,32]. However, GaN is not a laminar structure. The main reason for the decrease in hardness in 10 GaN is the decrease in relative density. From Figure 6, we can see that the relative density of 10 GaN-Cu decreased by 2% from 7.5 GaN-Cu, compared to a total decrease of 1% observed previously. The decrease in the relative density indicates an increase in the porosity of the composites, which is detrimental to the stiffness of the composites.

### 3.5. Electrical Conductivity Analysis of GaN-Cu Composites

**Figure 8:**
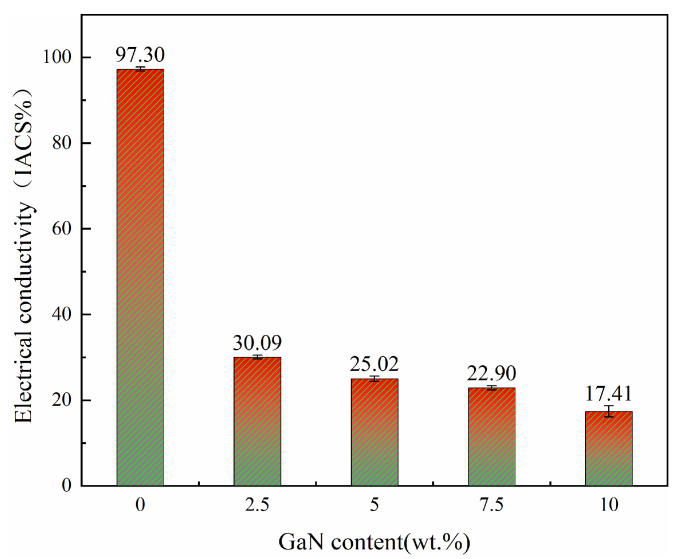
Relation between electrical conductivity and GaN mass fraction of sintered GaN-Cu composites.

Figure 8 shows the electrical conductivity of the GaN-Cu composite as a function of the GaN content. The trend in electrical conductivity is very interesting. With the addition of GaN, the electrical conductivity decreased sharply from 97.30% IACS of pure Cu to 30.09% IACS of 2.5 GaN-Cu. But as the content of GaN continued to increase from 2.5 wt%, the decrease in electrical conductivity became very slow. When the content of GaN increased by 5% from 2.5% to 7.5%, the electrical conductivity decreased only by 7.19% IACS. Overall, the electrical conductivity decreased precipitously and then became extremely slow; the descending line showed an “L” shape.

The addition of GaN leads to a decrease in electrical conductivity, with the result attributed to the fact that GaN is considered a semiconductor material. Cu, in turn, is a highly conductive metal, so when GaN is added to the Cu matrix, the GaN-Cu composites become a mixture of conductor and semiconductor; i.e., the electrical conductivity of the composites decreases [27,38]. The decrease in electrical conductivity in metal matrix composites is primarily attributed to conduction electron scattering, which arises from several factors. As reported in the literature, the key contributors to this phenomenon include (1) the increase in grain boundaries, which act as scattering centers for electrons [38]; (2) the obstruction of second-phase particles, which disrupt the free flow of electrons [39]; and (3) porosity, which introduces additional scattering sites and reduces the effective conductive pathways [28]. These factors collectively lead to a reduction in the electrical conductivity of the composites. Figure 8 shows that the addition of GaN has a more severe effect on the electrical conductivity of GaN-Cu composites. During sintering at 900 °C, the grains of pure Cu are free to grow unhindered, but the addition of GaN prevents the growth of Cu grains to the extent that a large number of grain boundaries are formed, which dominate the significant drop in electrical conductivity from pure Cu to 2.5 GaN-Cu. Starting at 2.5% GaN content, the change in GaN content has a relatively small effect on the electrical conductivity. This is possible as the grain boundaries of the composites are saturated and do not increase with GaN, but the increase in GaN occupies the position where copper transports electrons and hinders electron conduction, which dominates the slow decrease in electrical conductivity from 2.5 GaN-Cu to 10 GaN-Cu. In this work, the relative density of the composites varied over a small range, so the effect of pore space on conductivity was weak.

Although the conductivity of GaN-Cu composite materials monotonically decreases with increasing GaN content (from 30% IACS of 2.5 wt% GaN-Cu to 22.9% IACS of 7.5 wt% GaN-Cu), the 10 wt% GaN-Cu system exhibits maintained functional conductivity, maintaining the applicable threshold for advanced microelectronic interconnects or high-stress electrical contacts. This gradual decay is consistent with the inherent trade-off principle of metal matrix composites (MMCs) [3], where the excellent mechanical enhancement ability of GaN (through Orowan strengthening and load transfer mechanism [4]) requires a partial compromise in electron transfer efficiency.

## 4. Conclusions

In conclusion, GaN-Cu composites were successfully prepared by mechanical alloying with powder metallurgy, and it was demonstrated that GaN nanoparticles could improve the mechanical properties of metal matrix composites as reinforcement. The characterization of GaN-Cu indicates that GaN is crushed into nanoparticles and uniformly distributed in the copper matrix by ball milling without generating additional new material. The hardness increases and then decreases as the GaN content increases; 7.5 GaN-Cu has the highest hardness value of 128.66 HV, an increase of 130% compared to pure Cu. The electrical conductivity of GaN-Cu composites shows an “L”-shaped trend. The electrical conductivity has a significant decrease when GaN is added to the pure Cu matrix, while with the exponential increase in GaN content, the decrease in conductivity becomes very slow. Despite this, the prepared GaN-Cu composites still achieved an electrical conductivity of 22.9% IACS when the GaN content was 7.5%. These results show that GaN can be used as a reinforcement for copper matrix composites with significant potential applications for enhancement and possesses unique electrical properties.

In summary, while considering economic costs, research on GaN-reinforced copper-based composites represents valuable exploration in the field of multifunctional materials. Despite the trade-off between cost and performance, the unique properties and potential applications of GaN Cu composite materials, particularly in advanced microelectronics and hybrid electronic systems, demonstrate the necessity for continued research and optimization in these material systems.

## Data Availability

The original contributions presented in the study are included in the article, further inquiries can be directed to the corresponding author.
